# Positive Schizotypy Increases the Acceptance of Unpresented Materials in False Memory Tasks in Non-clinical Individuals

**DOI:** 10.3389/fpsyg.2020.00262

**Published:** 2020-02-21

**Authors:** Javier Rodríguez-Ferreiro, Mari Aguilera, Robert Davies

**Affiliations:** ^1^Grup de Recerca en Cognició i Llenguatge, Departament de Cognició, Desenvolupament i Psicologia de l’Educació, Universitat de Barcelona, Barcelona, Spain; ^2^Institut de Neurociències, Universitat de Barcelona, Barcelona, Spain; ^3^Department of Psychology, Lancaster University, Lancaster, United Kingdom

**Keywords:** schizotypy, semantics, false memories, confidence, metamemory, schizotypal personality

## Abstract

Enhanced spreading of semantic activation has been hypothesized to underlie some of the most significant symptoms of schizotypal personality, like thought disorder, odd speech, delusion, or magical thinking. We applied the Deese/Roediger-McDermott false memory task to the study of semantic activation in a group of 123 non-clinical individuals varying in the three dimensions of schizotypal personality: positive, negative and disorganized schizotypy. In the study phase, we presented them with lists composed of words semantically associated to unpresented critical words. Then, they responded to a recognition questionnaire including previously presented words and critical unpresented lures, as well as weakly related and unrelated unpresented lures. Participants rated their confidence in recognizing each word. They also filled in a standardized schizotypal personality questionnaire. Confirming the false memory effect, recognition ratings provided in response to critical words were higher than those produced for both weakly related and unrelated items. Crucially, scores in the positive dimension increased recognition percentages and confidence ratings for weakly related and unrelated lures. This study indicates that high levels of positive schizotypy might influence the tendency to accept false memories of unrelated unpresented material.

## Introduction

Schizotypal personality has been considered to be an indicator of vulnerability to psychosis (Meehl, 1962), based on markers such as eccentric behavior, strange speech, unusual beliefs, uncommon perceptive experiences or social isolation. When considering the presence of schizotypal traits as a state close to pathology, the study of such traits has usually been associated with clinical investigations that seek to deepen our knowledge about schizophrenia. However, from a dimensional perspective (Claridge, 1997), schizotypy is understood as a group of personality traits that are distributed continuously in the general population regardless of their relationship with the development of a pathology. In light of this approach, interest has grown in recent years in the study of the variability of cognitive abilities associated with the greater or lesser presence of schizotypal traits in non-clinical population (Aguilera-Ruíz et al., 2008; Mohr and Claridge, 2015).

Atypical patterns of semantic activation in individuals with schizotypal personality have been hypothesized to underlie some of the most significant schizotypal symptoms, like thought disorder (Johnston et al., 2008), odd speech (Minor et al., 2010), delusion (Laws and Bhatt, 2005), or magical thinking (Saunders et al., 2012). Empirically, this proposal is based on previous findings associating schizotypy with semantic effects such as more original responses in semantic fluency tasks (Kiang and Kutas, 2005; Rodríguez-Ferreiro and Aguilera, 2019) or increased semantic priming (Moritz et al., 1999; Johnston et al., 2008), among others. Theoretically, this hypothesis relies on a conception of semantic memory as a network of interconnected nodes in which, when a given node is activated, semantic activation spreads to associated nodes, with stronger associates receiving higher activation levels than weaker ones (Collins and Loftus, 1975). From this point of view, increased semantic activation spreading in schizotypal individuals, leading to over-activation of loosely associated concepts, could explain the appearance of atypical speech patterns with associative intrusions, as well as ideas of reference or magical ideation connecting unrelated events in high schizotypes (Mohr et al., 2001; Pizzagalli et al., 2001).

The Deese-Roediger-McDermott (DRM) paradigm (Roediger and McDermott, 1995) has also been used to explore the relation between schizotypy and semantic processing. In the DRM task, in the study phase, volunteers are presented with lists of words (e.g., “bed,” “rest,” “awake,” “tired,” “dream,” “wake,” “night,” “blanket,” “doze,” “slumber,” “snore,” “pillow,” “peace,” “yawn,” and “drowsy”) that each include words with a strong semantic relation with an unpresented critical word (the critical lure; e.g., “sleep”). Then, in the test phase, they are asked to recall the words presented and/or answer a recognition questionnaire indicating whether the critical lures, along with other presented and unpresented words, had been previously shown or not. In the original study, participants mistakenly recalled the critical words more than 40% of the time, and their acceptance rate in the recognition questionnaires was higher than 80% (Roediger and McDermott, 1995). The DRM task is theoretically relevant to the study of semantic processing because the false memory effect has been argued to result from the automatic associative activation of semantic information. Similarly to what happens during semantic priming tasks, in which semantic activation spreads from the prime to the target, enhancing its processing, semantic activation from items presented in the list spreads to the unpresented but semantically related critical lure, causing the false impression that it had, indeed, been presented (Hutchinson and Balota, 2005; Gallo, 2010).

Despite several previous attempts to clarify the relation between schizotypal personality and semantic processing, it remains uncertain whether schizotypy affects false memory rates or, if it does, whether one specific schizotypal dimension is responsible for that effect. Factor analyses-based studies have suggested that three dimensions can be distinguished within the schizotypy construct: positive, negative and disorganized (Kwapil and Barrantes-Vidal, 2015). The positive dimension includes symptoms that are not usually present in non-schizotypal individuals, like disruptions in the content of thought, magical thinking, extravagant perceptual experiences and paranoia. The negative dimension represents absence of normal functions, encompassing symptoms such as lack of will and motivation, lack of close friends, as well as anhedonia and flat affectivity. Finally, the disorganized dimension refers to atypical organization and expression of thought, unusual behavior and habits, as well as vague and elusive speech (Kwapil and Barrantes-Vidal, 2015). The traits associated to the different dimensions are related to each other, and significant correlations have usually been observed between these three schizotypal personality factors, specially between disorganization and the other two dimensions (Fonseca-Pedrero et al., 2015, 2018). Nevertheless, this three-factor solution, as opposed to unidimensional or bifactorial solutions, has been consistently supported in previous research (Raine, 1991; Raine et al., 1994; Fonseca-Pedrero et al., 2011, 2018). If false memory responses result from semantic memory processes (Reyna and Brainerd, 1995) and if schizotypy symptoms result from atypical patterns of semantic activation then, given the postulation of three (or more) latent dimensions underlying schizotypy symptoms, naturally the question arises: which schizotypy dimensions are associated with the production of false memory responses? This question is interesting, and the answers have potentially important theoretical implications, because identifying the association between variation in false memory responses and variation in one (or more) schizotypy dimensions will tell us how atypical semantic activation can result in schizotypal symptoms.

Although schizotypal personality has proven to be a useful construct to capture individual variability, it also presents problems due to a vague operationalization of its constituent dimensions based on various measurement instruments, often inconsistent with each other (Kwapil and Barrantes-Vidal, 2015). This problem extends to the study of the possible relationship between schizotypal personality and semantic processing, as previous studies aiming to clarify this issue have applied different assessment tools, some of which focus only on specific aspects of some of the schizotypal personality dimensions. The general hypothesis is that if schizotypal traits are caused by increased semantic activation, individuals with high scores in schizotypy questionnaires, specifically those measuring symptoms such as delusion, magic ideation, odd speech or thought disorder, should present higher false memory rates due to an overactivation of critical lures from presented words. So far, the evidence supporting this hypothesis is inconsistent and, in some cases, conflicting results have been observed even when the same assessment tools have been used. Previous studies investigating the association between false memories and schizotypal personality while administering full schizotypy are summarized in Table 1 [an extended summary can be accessed at the study folder in the OSF^1^ ].

**TABLE 1:** Summary of results of previous studies investigating the relation between false memories in the DRM paradigm and schizotypal personality.

Study	Scale used	Design	Relation with false memory rates	Dimension measured
Winogard et al., 1998	Dissociative Experiences Scale	Correlational	Positive	Positive
	VVIQ	Correlational	Negative	Positive
Laws and Bhatt, 2005	Peters Delusional Inventory	High vs. Low	Positive	Positive
Fisher et al., 2007	SPQ – Unusual Perceptive Experiences	Correlational	Null	Positive
	SPQ – Odd Beliefs	Correlational	Negative	Positive
	Chapman scales -Perceptual Aberration	Correlational	Null	Positive
	Chapman scales – Magical ideation	Correlational	Null	Positive
Dehon et al., 2008	Dissociative Experiences Scale	Correlational	Positive	Positive
	Peters Delusional Inventory	Correlational	Positive	Positive
Corlett et al., 2009	Chapman scales	Correlational	Null	Positive and negative
	Peters Delusional Inventory	Correlational	Null	Positive
Dagnall and Parker, 2009	SPQ-B – Cognitive-Perceptual	High vs. Low	Negative	Positive
	SPQ-B – Interpersonal	High vs. Low	Null	Negative
	SPQ-B – Disorganized	High vs. Low	Null	Disorganized
Meyersburg et al., 2009	Magical Ideation Scale	Correlational	Positive	Positive
	Tellegen Absorption Scale	Correlational	Positive	Positive
Sugimori et al., 2011	SPQ-B – Cognitive-Perceptual	Correlational	Null	Positive
	SPQ-B – Interpersonal	Correlational	Null	Negative
	SPQ-B – Disorganized	Correlational	Null	Disorganized
	LSHS	Correlational	Positive	Positive
	AHES-17	Correlational	Positive	Positive
Saunders et al., 2012	OLIFE-B – Unusual Experiences	High vs. Low	Positive	Positive
	OLIFE-B – Introvertive Anhedonia	High vs. Low	Null	Negative
	OLIFE-B – Cognitive Disorganization	High vs. Low	Null	Disorganized
	OLIFE-B – Impulsive Non-conformity	High vs. Low	Positive	impulsive non-conformity
Kanemoto et al., 2013	SPQ-B – Cognitive-Perceptual	Correlational	Null	Positive
	SPQ-B – Interpersonal	Correlational	Null	Negative
	SPQ-B – Disorganized	Correlational	Null	Disorganized
	LSHS	Correlational	Positive	Positive
	AHES-17	Correlational	Null	Positive
Hodgetts et al., 2015	OLIFE-B – Cognitive Disorganization	High vs. Low	Null	Disorganized

*VVIQ, Vividness of Visual Imagery Questionnaire; SPQ, Schizotypal Personality Questionnaire (B: Brief version); LSHS, Launay–Slade Hallucination Scale; AHES-17, Auditory Hallucination-like Experience Scale; OLIFE-B, Oxford-Liverpool Inventory of Feelings and Experiences – Brief version.*

In sum, previous studies have failed to provide a consistent pattern of results regarding the possible association between false memories and schizotypal traits. Those assessing the relation between false memories and positive symptoms have reported positive (Winogard et al., 1998; Laws and Bhatt, 2005; Dehon et al., 2008; Meyersburg et al., 2009; Asai et al., 2011; Saunders et al., 2012; Kanemoto et al., 2013), negative (Winogard et al., 1998; Dagnall and Parker, 2009), and null (Corlett et al., 2009; Sugimori et al., 2011; Kanemoto et al., 2013) associations between these two variables. Regarding the disorganized dimension, only one study has indicated a positive association with false memories (Saunders et al., 2012), with other four showing null associations (Dagnall and Parker, 2009; Sugimori et al., 2011; Kanemoto et al., 2013; Hodgetts et al., 2015). Finally, there appears to be no evidence associating the negative dimension and false memory rates (Corlett et al., 2009; Dagnall and Parker, 2009; Sugimori et al., 2011; Saunders et al., 2012; Kanemoto et al., 2013).

One possible explanation for the incongruences observed in previous studies addressing the association between schizotypal personality and false memories is that the former is not related with false memory rates, but with overconfidence in them (i.e., metamemory). According to Moritz and Woodward’s (2006) review, false memories in particular are not a differential feature of patients with schizophrenia. Nevertheless, these patients are characterized by a more liberal acceptance criteria, which leads them to display a reduced metacognitive awareness of their fallibility, as well as overconfidence in their errors. These tendencies would make them more prone to false beliefs and delusions.

Thus, it could be the case that schizotypal traits are not directly related to specific semantic-related memory processes, but to metacognitive awareness. The degree of confidence in one’s memories is of utmost importance because memories considered unreliable will probably be dismissed and will differentially affect our behavior compared to those we consider reliable (see Koriat and Goldsmith, 1998). In this sense, Laws and Bhatt (2005) showed that non-clinical individuals with high scores in a delusional ideation scale also displayed greater confidence for falsely recognizing unpresented items (both related and unrelated to presented words). Similarly, in Corlett et al.’s (2009) study, in which no significant relation was observed between schizotypal traits and false memory rates, positive symptoms were associated to higher confidence in the acceptance of unpresented words too.

Previous studies from the pattern recognition domain have observed that individuals showing greater belief in paranormal phenomena, which is considered to be a positive trait, tend to require a lower amount of objective evidence for the perception of meaningful patterns (Brugger and Graves, 1997). The results obtained by Brugger and Graves (1997) and other authors (e.g., Blackmore and Moore, 1994), support the hypothesis that individuals showing positive schizotypal traits present a Type I error bias which makes them more prone to perform positive identifications in the absence of compelling evidence. In our view, the overconfidence in the acceptance of unpresented materials observed by Laws and Bhatt (2005), Corlett et al. (2009) could be reflecting a similar mechanism.

Finally, the origin of the discrepancies between previous studies could also be related to a lack of control of certain characteristics known to influence false memories. In their review of emotion effects on false memory, Bookbinder and Brainerd (2016) argued that both the emotional content of the words and the volunteers’ mood during memory tasks are key aspects of false memory generation. On the one hand, negative content of the words, as measured by valence ratings (Bradley and Lang, 1994), has been shown to increase the false memory rates compared to neutral and positive content (Brainerd et al., 2008, 2010; Kanemoto et al., 2013). This observation has led to the hypothesis that negative content strengthens the processing of semantic relations among words (Bookbinder and Brainerd, 2016). On the other hand, enduring negative natural mood, like that presented in depression, has been shown to promote false memories (Moritz et al., 2005; Joormann et al., 2009). Following previous effects observed in relation to true memory, it has been suggested that enduring negative natural mood promotes a reliance on semantic-based memory traces as opposed to item-specific memory traces (Bookbinder and Brainerd, 2016). Schizotypal individuals present irregularities in emotional memory performance (Hoshi et al., 2011), and depression is known to be associated with positive (Lewandowski et al., 2006) and negative traits (Campellone et al., 2016) in schizotypy. Therefore, we believe that emotion-related effects should be accounted for when conducting experiments aimed to study the relation between false memories and schizotypy.

The aim of our study was to clarify the association between the different dimensions of schizotypal personality and semantic processing by means of a DRM task. The recognition questionnaire included target or presented words, critical or related unpresented words, weakly related unpresented words and unrelated unpresented words. We hypothesized that the volunteers should accurately recognize presented words based on both item-specific and semantic memory traces. Following spreading-of-activation interpretations of DRM effects (Gallo, 2010), we expected the participants to mistakenly recognize critical words on the basis of high levels of activation coming from the semantically related list words presented in the study phase of the experiment. Accordingly, we expected weakly related words to elicit lower recognition rates than critical items due to their dependence on weaker semantic associations to the presented words. Finally, unrelated words should be rejected by the participants due to the lack of activation supporting them. We expected to find a positive association between false memory of semantically related lures and positive symptoms, which would indicate that positive schizotypy is related to enhanced semantic activation. In order to control for a possible influence of emotion over false memory formation in our study, we took into account the valence and arousal of the words, and we introduced a measure of depression in our design.

Moreover, our recognition questionnaire consisted of a scale in which the volunteers had to state how confident they were that each word had or had not been presented. We could expect that if schizotypal personality is associated with differential metacognitive processes, then variability in the measures of schizotypal traits should be related with confidence in the recognition responses. Following previous studies (Laws and Bhatt, 2005; Corlett et al., 2009), we could expect increased confidence in the acceptance of unpresented words for individuals with high scores in the positive dimension of schizotypy, what could be interpreted as evidence of a Type I error bias.

## Materials and Methods

### Participants

A group of 123 Psychology students from the University of Barcelona (103 females, mean age = 19.98, *SD* = 3.25) took part in the experiment in exchange for course credits. They were all native speakers of Spanish or Spanish-Catalan native bilinguals. The university’s ethics committee (Comissió de Bioètica de la Universitat de Barcelona, CBUB) approved the study protocols (IRBOOOO3O99) and written informed consent was obtained from all the volunteers before their participation in the study. We treated all data anonymously.

Schizotypal personality was assessed by means of the Esquizo-Q-A questionnaire (Fonseca-Pedrero et al., 2009), which consists of 23 questions presented in a five points Likert-like format. The Esquizo-Q-A provides separate measures for the subscales Distortion of Reality (positive dimension, 6 items), which refers to positive symptoms such as distorted perceptive experiences, paranoid ideation and magical thinking, i.e., “I think there are people who can read other people’s minds”; Negative Dimension (negative dimension, 7 items), which refers to physical and social anhedonia, i.e., “I feel good when I see that my friends and family are happy” (negatively scored); and Interpersonal Disorganization (disorganized dimension, 10 items), which refers to symptoms like disorganized language and thinking, social anxiety and lack of close friends or weird behavior i.e., “People look at me funny because of my appearance”. We chose this questionnaire because it was constructed and validated for Spanish population. Scores in its three subscales have been shown to significantly correlate (Fonseca-Pedrero et al., 2009) with scores in the corresponding subscales of English-validated tests like SPQ-B (Raine and Benishaw, 1995) or RADS (Reynolds, 1987). We also used the Spanish version of the Beck Depression Inventory (BDI-II, Beck et al., 1996) as a control measure of enduring natural mood. This scale consists of 21 items with scoring scales ranging 0 to 3.

We present a summary of the scores of our participants in the personality and depression questionnaires in Table 2. Reliability values for the three dimensions of the schizotypy scale for our sample were acceptable for the positive dimension, α = 0.73, and poor for the negative and disorganized dimensions, α = 0.6.

**TABLE 2:** Summary and correlation between responses in schizotypy and mood questionnaires.

		Positive		Negative	Disorganized		BDI II
	Mean (*SD*)	9.35 (3.62) [9.12 (3.24)]		11.36 (2.95) [10.98 (2.49)]	25.82 (5.03) [25.73 (5.12)]		8.75 (6.64) [8.87 (6.82)]
	Min-max	6–27 [6–19]		7–23 [7–17]	11–37 [11–37]		0–28 [0–28]
Negative	r	0.091		–			
Disorganized	r	0.356 [0.431]	*** [***]	0.027	–		
BDI II	r	0.176 [0.268]	~ [**]	–0.038	0.488 [0.496]	*** [***]	–

∼*p* = 0.05, **p < 0.01, ***p < 0.001. Values within [] correspond to analyses excluding male participants.

### Experimental Task

The experiment was conducted with groups of up to ten volunteers per session, who were told that they were taking part in a memory test in which they would listen to lists of words. Before the experimental task started, the participants were asked to state their age and gender. Given the high prevalence of Spanish-Catalan bilinguals in the area, they were also asked to respond to a control question regarding their degree of proficiency in these two languages in a seven-point scale and confirm that they were native Spanish-speakers. Then the experiment started with the study phase of the false memory task, in which the participants listened to 18 lists of 12 words in one of two fixed pseudorandomized orders. The words were presented in a pre-recorded audio file at a rate of one word every 1.5 s. After each list, the participants had 1.5 min to write down all the words they could recall.

To construct the lists, we gathered backward associates (words that elicit a given response in free word-association tasks) for 18 critical lures selected from Spanish free association norms (Fernández et al., 2009). The norms provide proportions of individuals producing a critical word when prompted with a given word. This value that can be interpreted as an indicator of the degree of association between the two words. We selected the first 12 associates of each critical lure to be used in the study lists. For example, the list corresponding to “leader” consisted of the words “spokesperson,” “admired,” “idol”, “influence,” “champion,” “follower,” “initiative,” “protagonist,” “charism,” “fan,” “hegemony,” and “to command”. When the same word appeared in different lists, one of the repeated words was eliminated and a word from positions 13th or above was selected as a substitute. Following Roediger and McDermott (1995), we introduced a delay between the study and recognition phases of the experiment. The volunteers were asked to respond to the schizotypal personality questionnaire during this period.

For the recognition test, along with the 18 unpresented critical words (semantically related to the lists), we selected the words in the 1st (mean association to the critical word = 0.60, *SD* = 0.05) and 5th (mean association = 0.1, *SD* = 0.09) positions in each list to be presented as target (presented) words in the recognition test (e.g., “spokesperson” and “champion”). For each list, two not presented words from positions 13th and above of each list not appearing in any of the other lists were selected as weakly related lures (e.g., “admiration” and “quality”). Items in this category correspond to words for which, when presented in free association tasks, only a low percentage of individuals produce the critical word (mean association = 0.03, *SD* = 0.03). Finally, two unrelated words per list were also selected for the recognition test (e.g., “theory” and “snake”). For each of the 126 words used in the recognition questionnaire, we gathered valence and arousal data using values from Emofinder (Guasch et al., 2017), a web-based search engine for Spanish word properties from different normative databases (Redondo et al., 2005, 2007; Ferré et al., 2012; Guasch et al., 2016; Hinojosa et al., 2016; Stadthagen-Gonzalez et al., 2017). When data for any of the words did not appear in any of the databases (this was the case for six items) we used averaged values from twelve independent informants who filled in nine-point Likert-like scales for valence and arousal following the self-assessment manikin standard method (Bradley and Lang, 1994). The items used in the experiment are presented as Supplementary Material. We constructed two versions of the recognition questionnaire with two fixed pseudorandomized orders of item presentation, mirroring the procedure applied during the study phase. Following Laws and Bhatt (2005) the participants were asked to state how confident they were that each word had been presented in the study phase in a 1–4 scale where 1 stood for “Completely sure it has not been presented,” 2 stood for “Probably it has not been presented,” 3 stood for “Probably it has been presented” and 4 stood for “Completely sure it has been presented.” Hence, responses 1 and 2 indicated that the word had not been presented (rejection), whereas responses 3 and 4 indicated that the word had been presented (acceptance). After completing the recognition task, the participants responded to the natural mood questionnaire. The experimental session lasted around 45 min.

### Data Analysis

We analyzed the participants’ responses to each word category in the experimental task operationalized as recall percentages, recognition percentages, discriminability scores (derived from recognition percentages) and recognition confidence ratings. We started by analyzing the recall and recognition percentages corresponding to each word category in relation to the schizotypy measures by means of correlation analyses. Regarding recall, we analyzed the percentages of words produced by each participant which corresponded to target words (presented) and critical lures (not presented semantically related) as well as other unpresented words different from the critical lures. As for recognition, we started analyzing percentages of responses indicating that the word had been presented (aggregating responses 3 and 4 in the recognition questionnaire) for each word category.

Second, given that recognition percentages can be misleading because recognition responses depend on how liberal or conservative the participants are, we calculated three sensitivity scores for each participant following the signal detection theory approach (Stanislaw and Todorov, 1999). We calculated *d*’true values comparing hits to presented items with false alarms to unrelated unpresented items, as well as *d*’critical comparing the false recognition of critical items to false alarms to unrelated unpresented items, and *d*’weak, comparing the false recognition of weakly related items to false alarms to unrelated unpresented items. In order to correct our data when any of our participants presented zero hits or zero false alarms, which prevents the calculation of *d*’ scores, we followed the procedure recommended by Snodgrass and Corwin (1988). Thus, we calculated the hit rate as (number of hits + 0.5)/(number of presented items + 1), and the false-alarm rate as (number of false alarms + 0.5)/(number of unrelated-unpresented items + 1). The participants who are unable to discriminate between the respective item categories obtain *d*’-values close to zero, whereas those who tend to accept target words (or critical words in the case of *d*’critical and weakly related words in the case of *d*’weak) and reject unrelated unpresented lures obtain higher *d*’-values.

Third, we analyzed the confidence in the ratings separating between responses indicating that a given word had not been presented (responses 1 and 2) and those indicating that the item had been previously presented (responses 3 and 4). For this analysis, we recoded the responses so that those showing high confidence (i.e., responses 1 and 4: “Completely sure…”) were assigned 2 points, whereas those responses showing low confidence (i.e., responses 2 and 3: “Probably…”) were assigned 1 point. This way, we obtained separate measures for confidence in rejection and acceptance responses.

Fourth, in order to account for the possible influence of both item-level and participant-level valence-related effects in our participants’ responses, we further analyzed the data by means of a regression model including word valence and arousal values, as well as scores in the mood questionnaire, along with scores of the schizotypy questionnaire as predictors and confidence ratings as the dependent variable. Before starting the analyses, we standardized all predictors, including both item-level (valence and arousal) and participant-level (positive, negative, disorganized and BDI-II) variables. Standardizing continuous numeric predictors eliminates non-essential collinearity due to scaling (Cohen et al., 2003) and allows straightforward comparison of the effects. Condition number values (Belsley et al., 1980) were *k* = 1.99, indicating acceptable levels of interrelation between the predictor variables (values of *k* > 12 are indicative of collinearity according to standard criteria, Baayen, 2008).

Given the ordinal nature of our dependent variable, we analyzed the recognition ratings through cumulative link mixed models using the clmm2 function furnished by the ordinal package (Christensen, 2019). Cumulative link models can be understood to be generalizations of the logistic regression models used to analyze binary response variables (Agresti, 2002). In adopting this approach, the analysis involves fitting a model to estimate the effects of critical variables to influence (decrease or increase, on average), via a logit link, the probability that an observed response falls within a response category (when there are multiple ordered categories). The repeated measures nature of the response data requires a further distinction, between fixed and random effects, to allow an accurate accounting of error variance given the multilevel structure of the data (stemming from the repeated measures design) within the framework of generalized linear mixed-effects models. Mixed-effects models allow the researcher to estimate fixed replicable effects like those of word type or valence, as well as random effects such as unexplained effects due to random variation between items or participants.

We present the results of the model including the maximal random effects structure justified by our design (i.e., the structure including random intercepts and random slopes for all within-subjects and within-items experimental factors, Barr et al., 2013). Our final model included fixed effects corresponding to the main effects of word type (critical, target, weakly related, and unrelated) and the three schizotypy dimensions (positive, negative, and disorganized) as well as the interactions between the effects of word type and schizotypy dimension. Note that, in these kinds of analyses, effects of categorical variables such as word type are expressed as the comparison between a reference level, in our case critical words, to each other level. We also entered the valence and arousal values of the words and the participants’ BDI-II scores as covariates. The model incorporated random differences among sampling units both in intercepts and in the slopes of the fixed effects of word type, valence and arousal on participants and of positive, negative, disorganized and BDI-II scores on items.

## Results

All data are available from the OSF database^2^. Correlation analyses (see Table 3) conducted in JASP (JASP Team, 2019) showed no significant association between recall of any of the three word categories and schizotypal personality traits. Similarly, no significant associations appeared between scores in the schizotypal personality questionnaire and recognition percentages corresponding to critical lures or target words. In contrast, percentages of acceptance of unrelated and weakly related words were positively related with scores in the positive dimension. Finally (see Table 3), barely significant positive associations appeared between recognition percentages for weakly related stimuli and scores in the negative dimension (*p* = 0.042), and between recognition percentages for unrelated stimuli and scores in the disorganized dimension (*p* = 0.045).

**TABLE 3:** Summary of recall and recognition percentages and correlations with schizotypy measures.

	Mean (*SD*)	Positive r		Negative r		Disorganized r	
% Recall Critical Lure	1.88 (1.34) [1.972 (1.34)]	–0.012 [–0.016]		–0.146 [–0.018]		–0.094 [–0.038]	
% Recall Target	88.36 (4.51) [88.37 (4.67)]	0.017 [0.083]		0.012 [0.033]		0.155 [0.114]	
% Recall Other	9.72 (4.02) [9.66 (4.07)]	–0.015 [–0.090]		0.035 [–0.032]		–0.283 [0.091]	
% Recognition Critical Lure	69.98 (18.34) [71.54 (17.68)]	–0.094 [–0.050]		–0.144 [0.055]		–0.030 [0.080]	
% Recognition Target	80.34 (9.55) [80.33 (9.20)]	0.080 [0.174]		–0.070 [0.005]		–0.052 [0.027]	
% Recognition Weakly Related	22.58 (11.01) [22.69 (11.22)]	0.183 [0.273]	* [**]	–0.181 [–0.103]	* []	0.125 [0.135]	
% Recognition Unrelated	10.83 (9.46) [10.83 (9.9)]	0.238 [0.318]	** [**]	–0.087 [–0.094]		0.183 [0.194]	* [*]
d’ Critical	1.9 (0.59) [1.96 (0.56)]	–0.243 [–0.266]	** [**]	–0.064 [0.153]		–0.161 [–0.093]	
d’ True	2.2 (0.53) [2.21 (0.53)]	–0.148 [–0.159]		0.029 [0.112]		–0.214 [–0.189]	* []
d’ Weak	0.55 (0.41) [0.57 (0.42)]	–0.112 [–0.133]		–0.08 0.052		–0.112 [–0.131]	

*p < 0.05, **p < 0.01. Values within [] correspond to analyses excluding male participants.

**FIGURE 1:**
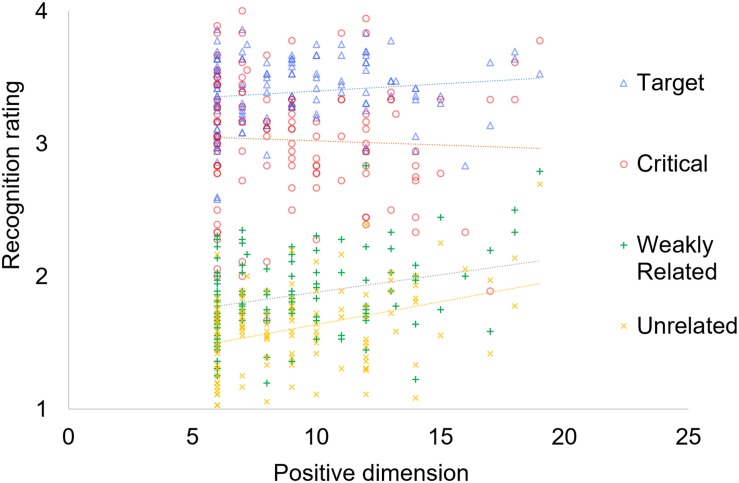
Summary of responses to the recognition questionnaire for each word category in relation to scores in the positive dimension.

A different set of correlation analyses including discrimination scores *d*’ and schizotypy measures indicated decreased discriminability for true items for participants with higher disorganized scores. Interestingly, we observed a significant negative correlation between discriminability of critical lures and scores in the positive dimension (see Table 3). This result was mainly mediated by an increase of false alarms for participants with high scores in this dimension (see Figure 1 below). As for confidence ratings, correlation analyses showed that individuals with higher scores in the positive and disorganized dimensions were less inclined to confidently reject weakly related and unrelated unpresented words (see Table 4).

**TABLE 4:** Summary of confidence for accepted or rejected words in each word category and correlations with schizotypy scores.

		CriticalLures	Target	WeaklyRelated		Unrelated	
**4.A Accepted words**							
	**Mean (*SD*)**	1.62 (0.23) [1.64 (0.22)]	1.82 (0.11) [1.82 (0.11)]	1.39 (0.25) [1.39 (0.25)]		1.12 (0.53) [1.13 (0.57)]	
Positive	r	–0.173 [–0.053]	–0.029 [0.100]	–0.029 [–0.054]		–0.137 [0.160]	
Negative	r	–0.126 [0.110]	–0.105 [–0.073]	–0.105 [–0.058]		–0.057 [–0.082]	
Disorganized	r	–0.010 [0.096]	–0.048 [0.011]	–0.048 [–0.101]		–0.148 [0.012]	
**4.B Rejected words**							
	**Mean (*SD*)**	1.36 (0.43) [1.35 (0.45)]	1.43 (0.27) [1.43 (0.28)]	1.59 (0.21) [1.6 (0.21)]		1.59 (0.24) [1.6 (0.24)]	
Positive	r	–0.137 [–0.142]	–0.021 [–0.038]	–0.189 [–0.194]	* [∼]	–0.292 [–0.246]	** [*]
Negative	r	–0.076 [–0.098]	0.090 [0.092]	–0.062 [–0.082]		–0.084 [–0.037]	
Disorganized	r	–0.121 [–0.073]	0.064 [0.081]	–0.294 [–0.248]	*** [*]	–0.237 [–0.225]	** [*]

∼*p* = 0.05, *p < 0.05, ** *p* < 0.01, and ***p < 0.001. Values within [] correspond to analyses excluding male participants.

The presence of an association between schizotypal traits and confidence ratings for weakly related stimuli led us to conduct a by-items examination of our results in order to ascertain whether the observed effects could be influenced by the inclusion of specific weakly related words in the analyses. Two of the weakly related items elicited mean confidence ratings higher than three (“*cansado*” and “*admiración*”), indicating that the volunteers were, on average, prone to mistakenly consider them as presented. New correlation analyses without these two items yielded the same results as those conducted with the full item set (confidence in rejection with positive dimension: *r* = –0.189, *p* = 0.036; with disorganized dimension: *r* = –0.284, *p* = 0.001), ruling out possible influence of specific characteristics of these items in the results.

Given that our sample mainly consisted of female participants, 84%, and taking into account that differences in the distribution of schizotypal traits between females and males have been observed in previous studies (Raine, 1992), we conducted complementary analyses excluding male participants from the sample. The general pattern of significant results remained unchanged (see values within brackets in Tables 2–4).

Regarding our regression analyses, we assessed whether the inclusion of random effects in our maximal model was justified by the data following a backward-selection heuristic (Matuschek et al., 2017) in order to fit a model with an adequate balance between power and conservatism. Both likelihood ratio test, χ^2^ (34) = 507.96, *p* < 0.001, and Akaike information criterion (AIC, Akaike, 1998) comparisons between the maximal model, AIC = 30900, and the same model without the random slopes, AIC = 31340, indicated that the maximal model provided a better fit to the data. Our analyses, see Table 5, indicated that recognition ratings provided in response to critical words were significantly lower than those corresponding to target words, but significantly higher than those produced for both weakly related and unrelated items. Crucially, a significant interaction appeared between word type and positive traits, showing that the effect of positive schizotypal traits was stronger for weakly related and unrelated words compared to critical items. Participants with higher scores in the positive dimension provided higher recognition ratings for the weakly related and unrelated items. In other words, although critical words were recognized falsely more often than weakly or unrelated words, this difference decreased for people high on the positive dimension (see Figure 1). No effects appeared in relation to the negative or disorganized dimensions. The analyses also indicated a significant negative effect of valence over recognition ratings for critical words, as more negative words received higher recognition ratings. The influence of BDI-II scores and arousal values over recognition ratings was not significant. These results confirm the association between scores in the positive dimension and recognition ratings provided to weakly related and unrelated words observed in the correlation analyses, when the three schizotypy dimensions are simultaneously taken into account, and ruling out possible influences of emotion-related variables.

**TABLE 5:** Summary of the cumulative linear mixed model.

Random Effects	Variance	*SD*			
Items (intercept)	0.882	0.939			
Positive	0.005	0.07			
Negative	0.002	0.05			
Disorganized	0.006	0.074			
BDI-II	0.007	0.083			
Participants (intercept)	0.907	0.952			
Critical vs. Target	0.783	0.885			
Critical vs. Weakly Related	0.613	0.783			
Critical vs. Unrelated	1.022	1.011			
Valence	0.017	0.132			
Arousal	0.003	0.051			

**Fixed Effects**	**Estimate**	**SE**	**z**	***p***	

Critical vs. Target	1.054	0.291	3.618	<0.001	***
Critical vs. Weakly Related	–2.488	0.287	–8.656	<0.001	***
Critical vs. Unrelated	–3.064	0.294	–10.437	<0.001	***
Positive	–0.144	0.105	–1.373	0.17	
Negative	–0.018	0.097	–0.184	0.854	
Disorganized	0.038	0.109	0.352	0.725	
Positive × Critical vs. Target	0.205	0.104	1.966	0.049	*
Positive × Critical vs. Weakly Related	0.273	0.097	2.831	0.005	**
Positive × Critical vs. Unrelated	0.387	0.113	3.431	<0.001	***
Negative × Critical vs. Target	–0.024	0.097	–0.242	0.809	
Negative × Critical vs. Weakly Related	–0.03	0.09	–0.33	0.741	
Negative × Critical vs. Unrelated	0.039	0.105	0.368	0.713	
Disorganized × Critical vs. Target	–0.086	0.105	–0.819	0.413	
Disorganized × Critical vs. Weakly Related	0.068	0.097	0.707	0.479	
Disorganized × Critical vs. Unrelated	0.04	0.113	0.355	0.723	
BDI–II	0.062	0.064	0.971	0.332	
Valence	–0.226	0.088	–2.562	0.01	*
Arousal	–0.117	0.086	–1.362	0.173	

*p < 0.05, **p < 0.01, and ***p < 0.001.

## Discussion

In this study, we aimed to clarify whether variability in schizotypal personality traits is associated with differences in semantic processing by means of a false memory experiment. We applied the DRM paradigm to a sample of non-clinical volunteers varying in schizotypy, also controlling for possible emotion-related effects. We included a measure of confidence in the responses in order to asses not only recognition percentages but also metacognitive processes. The percentages of recalled critical lures were generally low in our participants. In contrast, the expected false memory effect was more evident in the recognition questionnaire, as the participants tended to recognize target and critical words as presented but they tended to reject weakly related and unrelated items. Recall tasks have been shown to be less sensitive to false memory formation than recognition tasks (Roediger and McDermott, 1995), so it is unsurprising that recognition yields stronger effects in our experiment.

With regards to the association between false memory and schizotypal traits, in line with previous studies (Corlett et al., 2009; Dagnall and Parker, 2009; Sugimori et al., 2011; Saunders et al., 2012; Kanemoto et al., 2013), our results provided no support for an association between negative traits and false memory. So far, the strongest evidence of a relation between schizotypy and semantic memory linked positive traits with higher levels of false memories (Winogard et al., 1998; Laws and Bhatt, 2005; Dehon et al., 2008; Meyersburg et al., 2009; Asai et al., 2011; Saunders et al., 2012; Kanemoto et al., 2013), although several studies had also reported a negative association (Winogard et al., 1998; Dagnall and Parker, 2009), or lack of significant relation between these two variables (Corlett et al., 2009; Sugimori et al., 2011; Kanemoto et al., 2013).

In our experiment, we could not identify an effect of positive traits over the percentages of recall or recognition of the critical lures. Nevertheless, our analyses consistently showed that responses to weakly related and unrelated words were influenced by scores in the positive dimension. Volunteers with more pronounced positive traits presented higher percentages of false recognition for these word categories, producing higher recognition ratings than those with less marked traits. Indeed, the larger amount of unrelated words falsely recognized by higher scorers in the positive dimension was responsible for a decrease of discriminability between critical and unrelated lures in these participants in our signal detection analysis.

In our view, the fact that the effect of positive traits over false memory was not specific to false recognition of semantically related words indicates that it cannot be argued to reflect an association between schizotypal traits and enhanced semantic processing, so our initial hypothesis in this regard was not supported. It could also be argued that semantic effects observed in relation to schizotypal personality indicate not just enhanced or increased semantic activation, but that high schizotypes present broader or further-reaching semantic networks as some priming studies seem to indicate (Johnston et al., 2008; Rossell et al., 2014). This hypothesis could explain the increased ratings for weakly associated words in our results. However, the fact that, in our study, the effects appear not only in relation to weakly related words but also to unrelated ones, leads us to think that this is not the case.

Interestingly, our results point out that positive schizotypal traits influence the individuals’ confidence in their responses, in line with the results of other studies with non-clinical volunteers (Laws and Bhatt, 2005; Corlett et al., 2009) and with schizophrenia patients (Moritz and Woodward, 2006). Thus, according to our results, the relationship between schizotypal personality and false memory could be mediated by metacognitive processes: positive traits would be associated with metamemory mechanisms leading to higher confidence for false memories. Specifically, our confidence data suggested the possibility that high scorers in the positive dimension were less willing to reject weakly related and unrelated words. Such a tendency could be directly related to positive symptoms like delusions or paranormal beliefs, predisposing high schizotypes to magical thinking and superstition (Corlett et al., 2009). In this sense, our false memory and confidence results seem to indicate that individuals with positive schizotypal traits appear to be biased to accept memories for which no or little evidence of prior presentation is available. As we have already outlined in the introduction, in our view, this observation could reflect a general Type I error bias similar to that observed in studies of pattern identification, in which individuals with positive traits have been shown to be more prone to identify objects in noisy stimuli (Blackmore and Moore, 1994; Brugger and Graves, 1997). As for the disorganized dimension, one study had observed a positive association between this dimension and false memory (Saunders et al., 2012), whereas other studies failed to identify a significant relation between them (Dagnall and Parker, 2009; Sugimori et al., 2011; Kanemoto et al., 2013; Hodgetts et al., 2015). Our analyses showed significant correlations between disorganized traits and confidence ratings mirroring the results obtained in relation to the positive dimension, which is unsurprising given the significant degree of correlation between the scores in these two dimensions shown in our data and, consistently, in previous studies (Fonseca-Pedrero et al., 2015, 2018). Nevertheless, the fact that only the effects of the positive traits survived the regression model when the three dimensions, as well as valence-related control variables, were taken into account in the analyses, leads us to consider that positive traits play a more relevant role in the association between schizotypal personality and false memories.

Finally, although assessing the effects of emotion over false memory was not the main objective of this study, the fact that we controlled for emotion-related variables in our experiment allows us to draw relevant conclusions in this regard. Our data showed no significant effect of natural mood over the participants’ responses. The absence of effects in this regard could be due to a lack of variability in our non-clinical volunteers compared to previous studies in which patients with depression or post-traumatic syndrome had been assessed (Bookbinder and Brainerd, 2016). In contrast, we replicated previous findings regarding the influence of the emotional content of the words over false memories, as more negatively valenced critical lures, those with lower valence values, were associated to higher recognition ratings (Brainerd et al., 2008, 2010). This observation supports the claim that negative content strengthens the processing of semantic relations among words (Brainerd et al., 2008; Bookbinder and Brainerd, 2016), and adds to previous evidence for the semantic origin of valence effects on word processing (Kuperman et al., 2014; Rodríguez-Ferreiro and Davies, 2018).

Our results have real-life implications with regards to our understanding of mechanisms underlying the natural variability of personality features related to positive schizotypy. According to our data, a metacognitive bias influencing confidence in false memories could be associated to positive traits such as magical thinking and, thus, play a role, in the endorsement of phenomena like superstition or paranormal beliefs. Specifically, our analyses indicate that this mechanism influences judgments corresponding to materials for which none or very little evidence is present, linking our results to the hypothesis that individuals with positive traits present a Type I error bias. Our study sets the basis for future research investigating the nature of the relation between this tendency and magical thinking. For instance, studying whether an intervention aimed to reduce confidence in false memories could have an impact over the presence of paranormal beliefs could help determining if overconfidence plays a causal role in the development of these beliefs.

One limitation of our study is that our sample consisted exclusively of undergraduates. The recruitment of students for psychological experiments is common in the field. Among the studies we describe in the introduction, only one of them (Meyersburg et al., 2009) recruited participants outside the university campus (Hodgetts et al., 2015, did not report the origin of their sample). Nevertheless, recent studies have shown that student samples might not be fully representative of the general public specially in relation to personality characteristics (Hanel and Vione, 2016). Taking this into account, further studies should be conducted to ascertain whether our results are maintained in more representative samples of the general population.

Finally, another limitation of our study, related to the above, is that the sample was composed mainly by women. Although we have conducted analyses including only the female participants and the general pattern of results remained unchanged, our data cannot rule out possible gender-related influences in the interplay between schizotypal traits and false memories. Future studies with balanced samples should be conducted to clarify this issue.

## Data Availability Statement

The datasets generated for this study can be found in the OSF (https://osf.io/76ev2/).

## Ethics Statement

The studies involving human participants were reviewed and approved by the Comissió de Bioètica de la Universitat de Barcelona (CBUB – IRBOOOO3O99). The participants provided their written informed consent to participate in this study.

## Author Contributions

JR-F designed the study, supervised the data collection, and wrote the first draft. MA collaborated in data collection. JR-F and RD analyzed the data. JR-F, MA, and RD contributed to the manuscript revision, read, and approved it.

## Conflict of Interest

The authors declare that the research was conducted in the absence of any commercial or financial relationships that could be construed as a potential conflict of interest.

## Footnotes

1 https://osf.io/76ev2/

2 https://osf.io/76ev2/
